# Concurrent inferior vena cava syndrome and spontaneous retroperitoneal hemorrhage secondary to inferior vena cava filter thrombosis, treated with large bore mechanical thrombectomy: a case report

**DOI:** 10.1093/bjrcr/uaag024

**Published:** 2026-06-16

**Authors:** Basil Zia Khan, Andrew Wigham, Daniel Kearns

**Affiliations:** Department of Radiology, John Radcliffe Hospital, Oxford University Hospitals NHS Foundation Trust, Oxford OX3 9DU, United Kingdom; Department of Radiology, John Radcliffe Hospital, Oxford University Hospitals NHS Foundation Trust, Oxford OX3 9DU, United Kingdom; Department of Radiology, John Radcliffe Hospital, Oxford University Hospitals NHS Foundation Trust, Oxford OX3 9DU, United Kingdom

**Keywords:** thrombosis, inferior vena cava filter, retroperitoneal hemorrhage, large bore mechanical thrombectomy

## Abstract

Inferior Vena Cava (IVC) filters are an established treatment option to prevent morbidity and mortality in patients who are at high risk for pulmonary embolus and unable to receive pharmacological anticoagulation. As their use increases, we are now seeing an increasing incidence of associated complications; IVC thrombosis is one of them and can be challenging to treat. Our report describes a rare case of a 55-year-old male patient with active metastatic lung cancer presenting with concurrent inferior vena cava syndrome and spontaneous retroperitoneal hemorrhage due to an occlusive IVC filter-associated thrombus. The case demonstrates successful treatment with large bore mechanical thrombectomy. Our case aims to provide insight into a rare yet complex complication that can occur due to IVC filter use. Our hope is that this case increases awareness of how to successfully manage concurrent IVC filter thrombosis and resultant retroperitoneal hemorrhage with large bore mechanical thrombectomy.

## Summary

Inferior vena cava filter (IVCF) placement is a routinely performed procedure within interventional radiology. Current guidance suggests that an IVCF should be placed in patients with known venous thromboembolic disease and an absolute contraindication to anticoagulation.^1^ Several studies suggest an incidence rate of 2%-30% for IVCF-related thrombosis.^2^^,^^3^ Concurrent IVCF thrombosis and hemorrhage is extremely rare, with only a handful of cases reported in literature. We present a case of IVCF-associated thrombosis causing concurrent venous bleeding and IVC syndrome. We describe how this was diagnosed and successfully treated with large bore mechanical thrombectomy. This report also investigates current literature on such a rare occurrence.

## Clinical presentation

A 55-year-old male patient presented to the emergency department (ED) with collapse, severe periumbilical/left iliac fossa pain, and bilateral lower limb edema 4 days following resection of a brain metastasis from an Epidermal Growth Factor Receptor (EGFR)-positive lung adenocarcinoma. On examination there was rebound tenderness within the left iliac fossa, a palpable mass in the left lower quadrant and bruising. The patient was anticoagulated on apixaban for a previous pulmonary embolism. This had been withheld prior to neurosurgery in keeping with local protocols and an IVCF was placed (Cook Celect; Cook Medical). Post operatively the patient was started on a split treatment dose of Heparin based on weight as per our local protocol which was continued until presentation to the ED.

## Investigations/imaging findings

A CT Abdomen/Pelvis was performed to exclude/identify surgical causes. This was performed in the porto-venous phase as per our local protocols. The CT demonstrated a large left retroperitoneal hematoma measuring approximately 54 × 105 mm with an occlusive IVC thrombus extending below the filter to both common iliac veins (see Figure 1). There were dense foci within the hematoma which were equivocal for an active bleeding point given the absence of a multiphase study and no discrete vessel supply (see Figure 2). A second CT was performed a few hours later due to persistent hypotension despite adequate fluid resuscitation. This consisted of 3 phases (non-contrast, arterial and delayed) to increase the probability of identifying an active bleeding point. The repeat CT demonstrated no interval growth in the hematoma or active contrast extravasation. The patient continued to deteriorate clinically and became nonresponsive to fluid resuscitation. The hemoglobin level remained normal and did not drop. This was not in keeping with the extent of the hematoma. Given the out-of-hours presentation and short period of deterioration further imaging was not performed to assess the lower limb veins.

**Figure 1:**
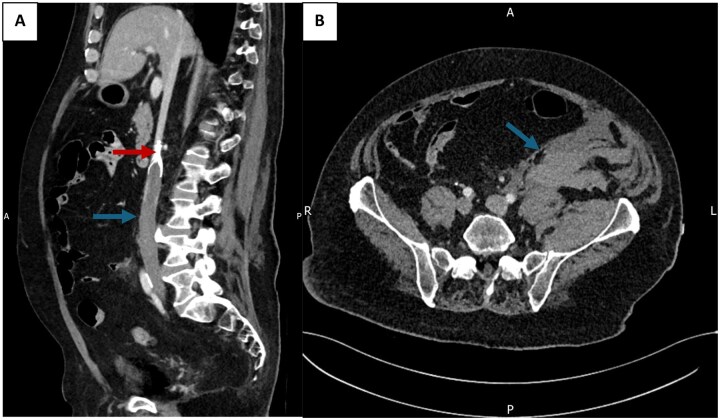
(A) demonstrates a porto venous phase sagittal CT image showing a difference in vessel attenuation and caliber above and below the IVCF. There is dilatation of the IVC below the filter (red arrow) and hypoattenuation (blue arrow). This is in keeping with an occlusive IVC thrombus (B) demonstrates an axial CT image in the arterial phase showing a large left retroperitoneal hemorrhage (blue arrow). This was noted to extend to both common femoral veins.

**Figure 2:**
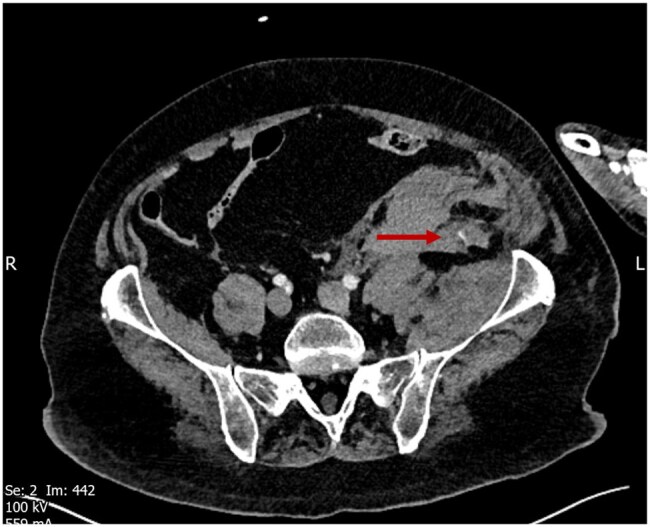
Axial CT demonstrating a focus of hyper density within the left retroperitoneal hematoma (red arrow). This was seen on the single phase porto-venous phase study performed at presentation. Given this was single phase study it was diAicult to confirm as a point of acute bleed. This finding was not replicated on subsequent Triple Phase CT performed a few hours later.

## Differential diagnosis

A multi-disciplinary approach involving the surgical, intensive care, hematological and interventional radiology teams was undertaken. Whilst initially the differential for hypotension and a retroperitoneal hematoma included an active arterial bleed it was later determined that the retroperitoneal hematoma was secondary to venous bleeding given the absence of an arterial blush or rapidly enlarging hematoma on serial imaging. This was felt to be the case due to increased venous backpressure from the occlusive IVC thrombus resulting in a perforated venous vessel. The persistent hypotension was felt to be due to a severe reduction in venous return to the heart resulting in obstructive shock/IVC syndrome. The reduced preload meant fluid resuscitation and vasopressor support were not effective in achieving hemodynamic improvement.

## Treatment

The presentation of concurrent hemorrhage and complete IVC thrombosis presented a dilemma in terms of treatment. Systemic or catheter directed thrombolysis was not suitable options given the bleed and recent neurosurgery. Surgical management would be extremely high-risk due anesthetic and operative risks. A joint decision was made to proceed with mechanical thrombectomy under conscious sedation. The rationale for selecting this treatment option was that thrombectomy would relieve the IVC obstruction therefore reducing venous hypertension and subsequently preventing further venous back bleeding. This would also allow increased return to the heart thus alleviating the obstructive shock. Complications of mechanical thrombectomy included vessel rupture/damage, pulmonary emboli and failure.

A 24 Fr DrySeal sheath (Gore) was inserted into the right common femoral vein (CFV) using ultrasound guidance. A total of 2500 units of heparin was administered during the procedure. The T24 FlowTriever system (Inari) was used for thrombectomy. A total of 6 aspirations were performed with excellent thrombus clearance and restoration of lower body venous return. There was immediate systolic blood pressure improvement from 90 to 150 mmHg. Post thrombectomy venograms demonstrated a patent IVC with prompt passage of contrast to the heart. No active contrast extravasation was seen to suggest an active bleed. After flow was re-established, a decision was made to leave the IVCF in situ and retrieve it at a later date due to an increased risk of clot propagation to the lungs. Closure of the CFV access site was achieved with a combination of manual compression and a purse string suture. Figure 3 highlights imaging from the procedure and a CT performed the following day. Hematology advice was sought regarding post procedural anticoagulation. A joint decision was made to continue split treatment heparin dosing based on patient weight as per our local protocols. The rationale being that the bleed occurred secondary to venous hypertension which had now been treated thus changing the risk/benefit ratio. The patient was felt to be more at risk of rethrombosis rather than a further bleed.

**Figure 3:**
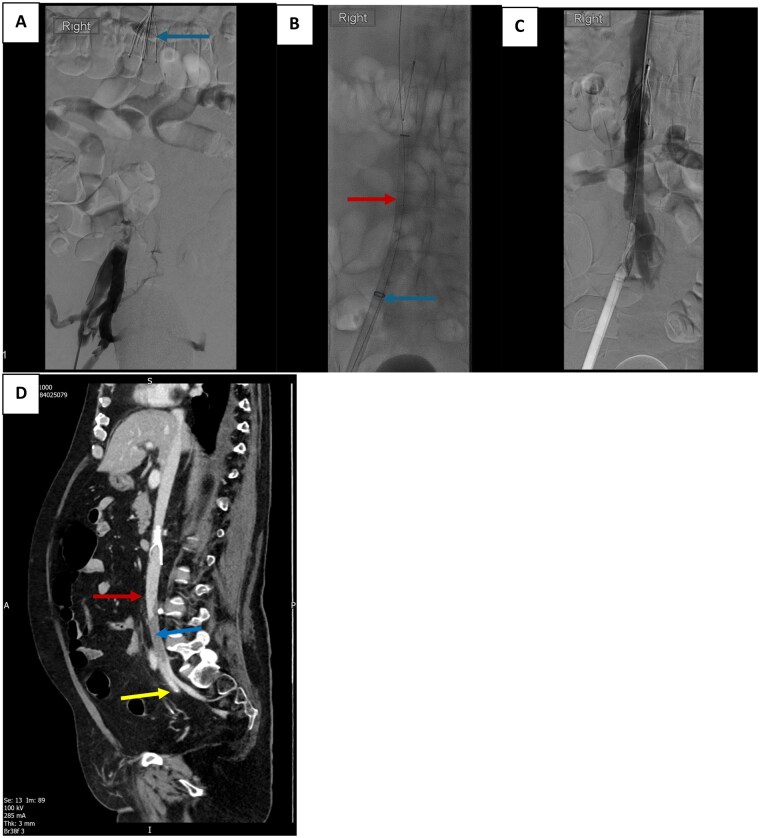
(A) Pre aspiration venogram demonstrating a long segment filling defect extending inferiorly from the IVC filter (blue arrow). This is in keeping with an occluded IVC. (B) fluoroscopic image showing a successfully deployed T24 Flowtreiver system within the IVC (red arrow) through a 24 Fr DrySeal sheath (blue arrow). (C) shows post pass venogram demonstrating restoration of flow through the IVC. (D) is a sagittal CT image in the porto-venous phase showing successful large volume removal of thrombus from the IVC (red arrow) with non-occlusive residual clots. (blue arrow). The Common Iliac veins are seen to opacify normally indicating patency (yellow arrow).

## Outcome/follow-up

The patient made a good recovery from this episode and was discharged from hospital 4 days later. Following 6 weeks of anticoagulation with split treatment dose Heparin, they successfully underwent removal of the IVCF. A CT performed 5 months following thrombectomy confirmed a patent IVC and resolution of the hematoma (see Figure 4). Unfortunately, the patient died 5 months later due to progressive metastatic disease.

**Figure 4:**
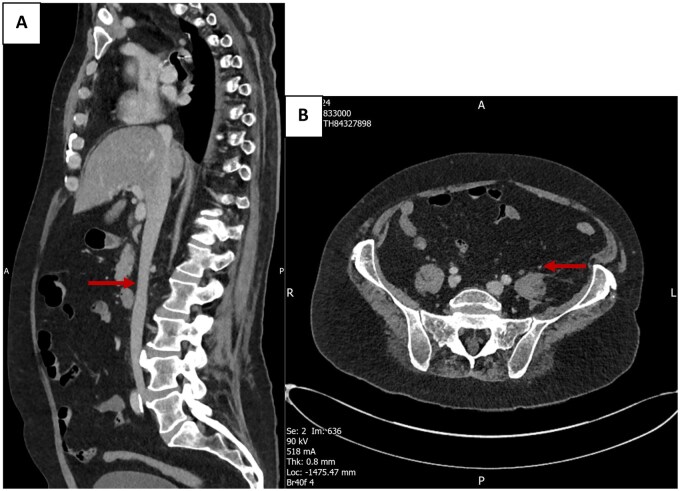
(A) sagittal CT performed 5 months following IVC thrombectomy showing a patent IVC with removed IVCF (red arrow). (B) show an axial picture demonstrating resolution of the left retroperitoneal hematoma (red arrow).

## Discussion

Complete occlusive thrombosis of IVCF resulting in obstructive shock is a rare complication. This alongside a concurrent retroperitoneal hematoma is even rarer. A comprehensive literature search of electronic databases (PubMed, Google Scholar) was performed using the keywords “IVC Filter,” “IVC Thrombosis,” “Hemorrhage” and “Mechanical thrombectomy” and demonstrated 3 relevant case reports where authors faced concurrent bleeding and IVCF related thrombosis.

The first is by Misono.^4^ The author describes a case of an IVCF-related thrombosis and subsequent caval rupture following initiation of therapeutic anticoagulation. Their patient had a long-term IVCF and symptoms of abdominal pain and bilateral lower limb swelling. Hypotension only occurred post-initiation of therapeutic anticoagulation rather than on presentation as seen in our case. The author proceeded with large bore mechanical thrombectomy using the 24 Fr FlowTriever System (Inari Medical) however they subsequently performed venoplasty across the filter and deployed an Abre self-expanding stent (Medtronic). It was their opinion that the long-term filter resulted in stenosis of the IVC which led to thrombosis resulting in the initial presentation. The author attributed the hemorrhage secondary to anticoagulation and increased venous back pressure. Unlike Misono, our patient had a much shorter IVCF indwelling period and did not have an IVC stenosis. We can hypothesize that in both cases occlusion of the IVC alongside anticoagulation predisposed patients to an increased bleeding risk due to higher venous back pressures and hypercoagulable blood.

The second relevant case is by Tseng et al^5^ The authors report that their patient presented with an IVCF-related thrombosis and a retroperitoneal hematoma. They were admitted with collapse and required cardiopulmonary resuscitation. CT confirmed a lumbar vein feeding into the hematoma and non-occlusive clots extending from the filter to the common iliac veins. Instead of opting for primary mechanical thrombectomy the authors performed embolization of the bleeding lumbar vein with immediate positive results. Despite stabilizing the hemoglobin and improving the hemodynamic status their patient reported of scrotal pain and left lower limb swelling 2 days following the embolization. This required treatment with mechanical thrombectomy using the AngioVac cannula and system (AngioDynamica). Whilst they retrieved significant clot, the long-term infrarenal filter was unretrievable. Their patient was commenced on Enoxaparin with a view to starting Warfarin; however, the patient was lost to follow up. A key difference between our cases is that Tseng et al’s patient suffered from hemorrhagic shock and had imaging evidence of a bleeding point. In contrast our patient was thought to be suffering from obstructive shock despite the presence of a hematoma. This was supported by the stable hemoglobin and size of hematoma on serial imaging. Between Tseng et al, our case and Misono we can hypothesize that IVCF-related thrombosis can lead to obstructive/hemorrhagic shock or a degree of both. We found that in cases of obstructive shock patients were less responsive to fluid resuscitation or vasopressor support as compared with hemorrhagic shock. This feature can help tailor whether to focus treatment on removing the obstruction (eg, vessel thrombosis) or stopping the bleed (eg, bleeding vessel).

Finally, Ludwig et al^6^ reported a case of concurrent IVC rupture and IVCF-related thrombosis. Their patient presented with severe atraumatic back pain, lower limb swelling and hematuria on a background of IVCF (Trap Ease, Cordis) insertion 3.5 years prior for recurrent DVTs. Ultrasound, CT and MR imaging confirmed a retroperitoneal hematoma and thrombosis of the IVC extending to the femoral veins. Despite a mild reduction in hemoglobin the authors opted for conservative treatment in view of hemodynamic stability (stopped concurrent anticoagulation). CT imaging at 3 and 4 months revealed a shrinking hematoma. They report that the lower limb swelling whilst initially worsened improved with medical management (furosemide). They successfully restarted the patient on therapeutic anticoagulation 4 months following admission. This case differs from all 3 by means of management. Whereas all our patients share similar pathologies the range in severity highlights the unpredictability of IVCF-related complications. This emphasizes the need to investigate, diagnose and treat based on clinical history, disease progression and imaging findings.

Table 1 has been adapted and adjusted from Gao et al.^7^ This summarizes current reports where authors have successfully treated obstructive shock from extensive IVCF thrombosis with mechanical thrombectomy. Alongside our report, each of these cases adds further to the increasing literature on the safety and effectiveness of mechanical thrombectomy when dealing with acute thrombosis. Table 1 shows that patients often present with general symptoms including abdominal pain, lower limb swelling or hypotension. In the absence of good clinical history diagnosis can be challenging often requiring detailed interpretation of the CT. It is important to highlight that in a critically unwell patient with an IVCF, the radiologist must pay special attention to the filling/opacification of the IVC and iliac veins for signs of thrombosis/occlusion.

**Table 1:** Case reports on obstructive shock secondary to IVC syndrome in patients with a IVCF treated with mechanical thrombectomy–adapted and adjusted from Gao et al^7^.

Study	Presentation	IVCF Details	Imaging Findings	Intervention	Outcomes	Anti coagulation
**Gao et al^7^**	Abdominal pain, bilateral lower limb swelling and hypotension	Inserted post trauma 5 days prior to presentation.	CTV -Thrombosis of IVC extending below filter to bilateral common femoral veins and distal lower limbs veins	Angiojet Thrombectomy System (Boston Scientific)	IVC patency resorted however second thrombectomy procedure performed due to thrombosis. CT at 3 months post op showed patent IVC and iliacs.	Post operatively on continuous heparin infusion however discharged on Apixaban 2.5 mg twice a day
**Mohammed et al^8^**	Confusion, bilateral lower limb swelling and hypotension.	Inserted 15 years prior following trauma.	US, CT and invasive venography confirmed Thrombosis of IVC extending to bilateral femoral veins.	Mechanical Thrombectomy—device not mentioned.	350 ml of thrombus cleared—approximate. Hemodynamic status stabilized immediately. IVCF not removed. Patient asymptomatic and 3 months follow up.	Unfractionated heparin post op later switched to lifelong Rivaroxaban.
**Lane et al^9^**	Sudden onset abdominal pain and hypotension	IVCF inserted 12 years ago without retrieval	CT showed occlusive thrombosis of IVC extending from filter to bilateral common iliac veins. US showed thrombus in right lower limb veins	ClotTreiver and FlowTreiver (Inari Medical)	CT 1 day post procedure demonstrated retroperitoneal and right obturator muscle hematomas presumed to be due to anticoagulation managed with transfusion. US at 4 months revealed patent vessels. At 6 months patient was asymptomatic	Discharged on long term Apixaban.

It is interesting to note that most cases in current literature report long-term IVCFs. We find that only Gao et al^7^ and our case demonstrate a short history from IVCF insertion to presentation with acute obstructive shock.

A retrospective review by Li et al^10^ investigated the outcomes of 15 acutely unwell patients with symptomatic IVCF-related thrombosis undergoing large bore mechanical thrombectomy. They reported a technical success rate of 73% with 100% improvement in symptoms. The 30-day mortality rate was 13% (1 intra-procedural death due to PE and 1 post-operatively due to malignancy complications). Similar to our case Li et al’s review demonstrates that large bore mechanical thrombectomy is a safe and effective option for treating occlusive IVCF-related thrombosis.

In conclusion, IVCF-related thrombosis and concurrent hemorrhage are rare occurrences presenting both a diagnostic and treatment challenge. We hope our report provides an example of how this can be successfully diagnosed and treated.

## Data Availability

Not applicable.

## Learning points

- Occlusive IVCF thrombosis can result in obstructive or hemorrhagic shock due to a combination of reduced return to the heart and back pressure venous bleeding.
- Careful interpretation of CT is key to diagnosing IVCF thrombosis.
- Combination of bleeding and thrombosis in a shocked patient can present a dilemma in terms of treatment as observation, surgery or thrombolysis/anticoagulation are not safe options.
- Endovascular options including large bore mechanical thrombectomy have proven safe and effective in such presentations.
